# Editorial: Nutrition, Immunity and Viral Infections

**DOI:** 10.3389/fnut.2020.00125

**Published:** 2020-09-08

**Authors:** Julio Villena, Takeshi Shimosato, María Guadalupe Vizoso-Pinto, Haruki Kitazawa

**Affiliations:** ^1^Laboratory of Immunobiotechnology, Reference Centre for Lactobacilli, National Council of Scientific and Technological Research, San Miguel de Tucumán, Argentina; ^2^Food and Feed Immunology Group, Laboratory of Animal Products Chemistry, Graduate School of Agricultural Science, Tohoku University, Sendai, Japan; ^3^Department of Biomolecular Innovation, Institute for Biomedical Sciences, Shinshu University, Nagano, Japan; ^4^Infection Biology Lab, Instituto Superior de Investigaciones Biológicas, National Council of Scientific and Technological Research - National University of Tucuman, San Miguel de Tucumán, Argentina; ^5^Laboratorio de Ciencias Básicas (Genética), Facultad de Medicina, Universidad Nacional de Tucumán, San Miguel de Tucumán, Argentina; ^6^Livestock Immunology Unit, International Education and Research Centre for Food and Agricultural Immunology, Graduate School of Agricultural Science, Tohoku University, Sendai, Japan

**Keywords:** virus infection, immunity, immunobiotics, nutritional immunology, mucosal immmunity

Viral infectious diseases have a great impact on humankind. Pandemic, epidemic, and endemic viral diseases produce considerable morbidity and mortality, negatively affecting not only health and well-being but also local and global economies by increasing school and work absenteeism as well as the healthcare system expenses. Probably the best example of this global threat is the infectious disease caused by the novel Severe Acute Respiratory Syndrome Coronavirus 2 (SARS-CoV-2), which has infected millions of people globally during the 2019-2020 pandemic [WHO, coronavirus pandemic; (1)]. Viral infections not only affect the economy in terms of human life, they also induce losses in livestock and crops (2), and can break down the barriers between animals and people, creating new potential dangers to human health (3). The SARS-CoV-2 pandemic pushed healthcare systems around the world to the limit and put pressure on the scientific community to provide solutions that help to prevent or alleviate its harmful effects. In consequence, in the past few months, there has been a reevaluation of the work of scientists actively investigating the biological features of viral infections, as well as potential preventive and therapeutic tools to combat them.

As a discipline, Nutritional Immunology is working actively, contributing to the prevention of viral infections (4–7). One of the most important fields of Nutritional Immunology is the study of the relationship between nutrition, immunity, and infections. During recent decades, incredible advances have been made in understanding how nutrients (or the lack of them) influence the microbiota and the immune system and affect resistance to viral infections. Scientists have gained insight into the cellular and molecular interactions of nutrients and microorganisms with the immune system, and this information has allowed the development of practical applications and biotechnological tools for improving the immune system and ameliorating the negative consequences of viral infections in humans and animals. The manuscripts gathered in this Research Topic are examples of the mechanistic and applied investigations into the effects of nutritional and immunological interventions on viral diseases.

The interaction and stimulation of intestinal epithelial cells (IECs) and antigen presenting cells (APCs) in the gastrointestinal mucosa by beneficial immunomodulatory microorganisms have been suggested as one of the most important mechanisms involved in the improvement of the resistance against viral infections induced by nutritional and immunological interventions with probiotic foods and feeds (1, 4, 8). Therefore, there is great interest in elucidating the mechanisms involved in the interaction between beneficial microorganisms with IECs and APCs, which are the first to meet the microbes and their molecules reaching the intestinal mucosa. In this Research Topic, Garcia-Castillo et al., Kanmani and Kim, and Albarracin et al., provide some clues of the cellular and molecular mechanisms involved in the beneficial interactions of probiotic lactobacilli strains with IECs and APCs. Interestingly, Albarracin et al., have demonstrated that the interaction of probiotic lactobacilli such as *Lactobacillus rhamnosus* CRL1505 or *L. plantarum* MPL16 with IECs not only influences the antiviral immune response in the gastrointestinal tract but, in addition, may contribute to the beneficial modulation of the innate antiviral responses in distant mucosal tissues such as the respiratory tract.

In addition to studying the probiotic-induced immune changes in the host, it is also necessary to find out which bacterial molecules are responsible for their beneficial effects. In this regard, Mizuno et al. developed a D-alanyl-lipoteichoic acid biosynthesis protein knockout-mutant strain, to demonstrate the key role of lipoteichoic acid in the anti-inflammatory effect induced by the probiotic *L. plantarum* CRL1506 strain in the context of Toll-like receptor (TLR)-3-mediated intestinal inflammation. The characterization of the immunomodulatory effects of beneficial microorganisms and their effector molecules on the mucosal immune system provides a scientific basis to apply them for modulating both the innate immunity and the adaptive immunity targeting specific antigens. Thus, microorganisms and immunomodulatory molecules have been proposed as adjuvants for the generation of mucosal vaccines. Raya Tonetti et al. demonstrated that bacterium-like particles (BLPs) obtained from different immunomodulatory lactobacilli strains differed in their abilities to regulate intestinal and systemic adaptive immune responses induced by the oral administration of a rotavirus vaccine. The work proposed that BLPs derived from highly immunomodulatory lactobacilli strain as an excellent alternative for the development of mucosal antiviral vaccines, indicating that it is necessary to appropriately select BLPs to find those with the most efficient adjuvant properties. In addition, Nigar and Shimosato have reviewed how unmethylated cytosine–guanine dinucleotide (CpG) motifs and single-stranded synthetic oligodeoxynucleotides, acting through TLR9 activation, are potent stimulators of the host immune response making them an interesting alternative as mucosal adjuvants for antiviral and antitumor vaccines.

Nutrients such as micronutrients and flavonoids also have been shown to influence the immune responses against viral infections. In this regard, zinc has been shown to regulate diverse physiological functions and to play crucial, and sometimes divergent roles in viral infections. Kar et al. demonstrated that zinc depletion inhibited Dengue Virus and Japanese Encephalitis Virus infections in IECs but had no effect on rotavirus infection. These results pointed out that modulation of zinc homeostasis during virus infection could be a component of the host antiviral response. Thus, the modulation of zinc homeostasis could be used as a potent antiviral strategy against flaviviruses. On the other hand, Wu et al. reported that puerarin, an isoflavonoid isolated from the traditional Chinese herb Gegen, differentially modulates the innate immune response against the Porcine Epidemic Diarrhea Virus. The proteomic study performed in this work both in cell cultures and in neonatal pigs demonstrated the ability of puerarin to inhibit the virus-induced nuclear factor (NF)-κB activation and inflammatory damage as well as to increase the expression of several interferon (IFN)-stimulated genes. These two original research articles are examples of how Nutritional Immunology applied to viral infections can offer the scientific basis for the development of new antiviral foods and feeds.

On the other hand, it is well-known that the adipose tissue plays key roles in immunometabolism in health and disease conditions such as viral infections. Therefore, investigating the ability of cells in the adipose tissue to respond to microbial ligands may contribute to a better understanding of the role of this physiological system in resistance to infections. It was reported that human and mouse adipocytes are capable of responding to TLR3 activation by producing tumor necrosis factor (TNF)-α, interleukin (IL)-6, IL-8, and chemokine C-C motif ligand (CCL)-2 as well as IFN-α/β and multiple anti-viral proteins (9, 10), indicating that adipose cells are able to trigger innate antiviral responses. In this Research Topic, Igata et al. performed a global transcriptomic study in porcine intramuscular mature adipocytes following the stimulation with TLR2, TLR3, and TLR4 ligands. Interestingly, the work demonstrated that porcine adipocytes, similar to human and mouse cells, can respond to TLR3 activation by increasing the expression of several genes (*CCL2, CCL8, CCL5, CCL3L1, IL1*β, and *IL12*) that participate in antiviral inflammatory responses. This work opens the doors for future research on the response of adipocytes in the defense of viral infections in the porcine host, as well as its potential use as a human model.

Interestingly, zebrafish has become a well-recognized animal model to study host-microbe-immune interactions because of the diverse set of laboratory tools available for these cyprinids, including the possibility of generating germ-free individuals or the *in vivo* imaging of specific immune cell populations in whole transgenic organisms. López Nadal et al. revised the practical advantages of zebrafish and discussed how this model sheds light on the mechanisms by which feed influences host-microbe-immune interactions and ultimately fish health and resistance to infections. As an interesting example of the use of the zebrafish model for the study of immunomodulatory feeds Ikeda-Ohtsubo et al., reported the capacity of a fucose-rich sulfated polysaccharide called fucoidan, extracted from edible seaweed *Cladosiphon okamuranus*, to modulate microbiota and immune responses.

The collection of reviews and original research articles presented under this Research Topic provide a comprehensive set of information on the potential of nutritional interventions to beneficially modulate antiviral immune responses in both humans and animals. The editors hope that this topic will act as a potent stimulus for further research in this growing and exciting area of the Nutritional Immunology.

## Author Contributions

All authors listed have made a substantial, direct and intellectual contribution to the work, and approved it for publication.

## Conflict of Interest

The authors declare that the research was conducted in the absence of any commercial or financial relationships that could be construed as a potential conflict of interest.
